# Imaging features of invasion and preoperative and postoperative tumor burden in previously untreated glioblastoma: Correlation with survival

**DOI:** 10.4103/2152-7806.68337

**Published:** 2010-08-10

**Authors:** Rohan Ramakrishna, Jason Barber, Greg Kennedy, Adnan Rizvi, Robert Goodkin, Richard H. Winn, George A. Ojemann, Mitchel S. Berger, Alexander M. Spence, Robert C. Rostomily

**Affiliations:** 1 Departments of Neurological Surgery, University of Washington, Seattle, US; 2 Departments of Neurology, University of Washington, Seattle, US; 3 Department of Surgery, University of Wisconsin, Madison, US; 4 Department of Surgery, Oregon Health Sciences University, US; 5 Department of Neurosurgery, Mt. Sinai School of Medicine, New York, US; 6 Department of Neurological Surgery, University of California, San Francisco, US

**Keywords:** Glioblastoma, invasion, MR imaging, resection, survival, tumor burden

## Abstract

**Background:**

A paucity of data exists concerning the prognostic usefulness of preoperative and postoperative imaging after resection of glioblastoma multiforme (GBM). This study aimed to connect outcome with imaging features of GBM.

**Methods:**

Retrospective computer-assisted volumetric calculations quantified central necrotic (T0), gadolinium-enhanced (T1) and increased T2-weighted signal volumes (T2) in 70 patients with untreated GBM. Clinical and treatment data, including extent of resection (EOR), were obtained through chart review. T1 volume was used as a measure of solid tumor burden; and T2 volume, as an indicator of invasive isolated tumor cell (ITC) burden. Indicators of invasiveness included T2:T1 ratios as a propensity for ITC infiltration compared to solid tumor volumes and qualitative analysis of subependymal growth and infiltration of the basal ganglia, corpus callosum or brainstem. Cox multivariate analysis (CMVA) was used to identify significant associations between imaging features and survival.

**Results:**

In the 70 patients studied, significant associations with reduced survival existed for gadolinium-enhancing tumor crossing the corpus callosum (odds ratio, 3.14) and with increased survival with gross total resection (GTR) (GTR median survival, 62 weeks versus 37 and 34 weeks for sub-total resection and biopsy, respectively). For a selected “GTR-eligible” subgroup of 52 patients, prolonged survival was associated with smaller preoperative gadolinium-enhancing volume (T1) and actual GTR.

**Conclusion:**

Some magnetic resonance (MR) imaging indicators of tumor invasiveness (gadolinium-enhancing tumor crossing the corpus callosum) and tumor burden (GTR and preoperative T1 volume in GTR-eligible subgroup) correlate with survival. However, ITC-infiltrative tumor burden (T2 volume) and “propensity” for ITC invasiveness (T2:T1 ratio) did not impact survival. These results indicate that while the ITC component is the ultimate barrier to cure for GBM, the pattern of spread and volumes of gadolinium-enhancing solid tumor are more robust indicators of prognosis.

## INTRODUCTION

Imaging abnormalities detected on standard MR imaging studies for patients with untreated glioblastoma multiforme (GBM) include peripherally increased T2 signal, uptake of gadolinium, and centralhypodensities that correspond to vasogenic edema in brain infiltrated with isolated tumor cells (ITCs), solid tumor with blood-brain-barrier disruption and necrotic cyst formation, respectively.[23–25] The volumes and anatomic locations of these MR imaging abnormalities vary considerably among patients with GBM at the time of diagnosis, but it is unclear whether variability in these imaging components reflects phenotypic differences in tumor biology that relate to outcome. Thus, we propose to test the hypothesis that variability in abnormal MR imaging features related to tumor invasiveness and/or tumor burden predict patient outcome.

The correlation between MR imaging features and distinct histopathologic features allows analysis of imaging studies to provide indirect quantification of a tumor’s histopathologic composition. For instance, studies that have analyzed brain tissue samples from radiographically defined regions in GBM patients demonstrate that contrast enhancement and edema are good indirect markers for the presence of solid tumor tissue with neovascularity and invasive isolated tumor cells, respectively, while areas of central hypodensity represent tumor necrosis.[23–25] Additional information about invasiveness can be derived from other MR-evaluable indicators, such as subependymal contrast enhancement, edema, contrast enhancement in the contralateral corpus callosum, or tumor extension into the brainstem or basal ganglia, which reflect the known propensity for GBM cells to migrate along white matter tracts.[15,30,39] Thus, quantitative and qualitative characterizations of the extent and distribution of MR imaging abnormalities in untreated GBMs are expected to provide a good measure of tumor infiltration and burden.

Previous analyses of the correspondence between GBM imaging characteristics and survival focused on preoperative and postoperative contrast-enhancing tumor volumes.. These studies generally report no association between preoperative tumor volume or burden and survival. Smaller postoperative enhancing tumor volume tends to correlate with improved patient outcome, but this remains controversial.[2,3,16,17,22,26,27,28,32,42,44] In contrast, few reports have analyzed the contribution of tumor edema or ITC burden to outcome,[16] or have attempted to comprehensively characterize features of tumor invasiveness and relate these to patient outcome.[37] The present analysis was undertaken to determine whether indicators of invasiveness or estimates of invasive or solid tumor burden might provide additional means by which to stratify patients prognostically.

## PATIENTS AND METHODS

### Patient characteristics and clinical data acquisition

Adult patients (>18 years old) treated at the time of initial pathological diagnosis of GBM at the affiliated University of Washington hospitals were identified through a comprehensive medical records search. Criteria for inclusion were as follows: no prior therapy or cytoreductive surgery, availability of pre-treatment imaging studies (T1-weighted gadolinium-enhanced and T2-weighted images) with gadolinium-enhancing tumors, postoperative CT scans with and without contrast within 48 hours of surgery, postoperative treatment with a standard course of postoperative radiation therapy (≥ 59.4 Gy), and histopathological confirmation of GBM. The criteria used for the microscopic diagnosis of GBM included the presence of necrosis. Seventy patients fulfilled these inclusion criteria. Follow-up was complete (to expiration) for 66 of the 70 patients. The remaining 4 patients were followed for 104, 208, 230 and 230 weeks, respectively, until lost to follow-up.

All patients received adjuvant radiation therapy (>5940 Gy) except one who died of disease progression during radiation therapy (XRT). Of the 70 patients, 42 received adjuvant chemotherapy. Of those who received chemotherapy, all received alkylating agents alone or as part of combinatorial therapy that often included carboplatin. As multiple chemotherapeutic approaches were used during the period of the study, the small numbers in each subgroup did not allow for meaningful analysis of the effect of specific chemotherapy on outcome.

Data on patient characteristics, treatment histories and survival were collected from hospital records, outpatient clinic notes and tumor board summaries. All patients were followed by the University of Washington Neuro-Oncology Tumor Board. Treatment data included the extent of surgical resection, steroid use prior to MR imaging and the number of subsequent therapies (radiation, chemotherapy or reoperation). A summary of the patient characteristics is provided in Table 1.

**Table 1:** Patient and imaging characteristics

Age (mean) (range)	58	(22-79)
Sex (M/F)	51/19	
Preoperative KPS (≥70)	58	83%
Chemotherapy Postop.	58	83%
Total # Other Therapies (mean) (range)	0.9	(0-3)
Primary Tumor Location		
Lobar	58	83%
Mixed/Deep	12	17%
Extent of resection		
Biopsy	7	10%
Sub-total resection	31	44%
Gross total resection	32	46%
Involvement of basal ganglia		
None	30	43%
Edema only	26	37%
T1 Gadolinium enhancement	14	20%
Involvement of contralateral corpus callosum		
None	55	79%
Edema only	5	7%
T1 Gadolinium enhancement	10	14%
Involvement of brainstem		
None	61	87%
Edema only	6	9%
T1 Gadolinium enhancement	3	4%
Subependymal contrast enhancement	23	33%
Tumor volumes (cm^3^) and ratios; (mean) (range)		
T0	5.8	(0-53.1)
T1	36.8	(2.7-122)
T1 total	42.6	(2.7-145)
T2	69	(0.9-378.6)
T2 total	111.6	(4.9-448.6)
T2/T1	2.8	(0.2-16.0)
T2/T1 total	2.6	(0.1-13.5)

KPS: Karnofsky performance score; T0: necrotic volume; T1: gadolinium-enhancing volume - T0: necrotic cyst volume; T1 total: T1±T0; T2: total increased T2 signal volume minus (T1 ± T0; T2 total: (T0±T1±T2)

### MRI data collection

#### Terms and definitions

**Figure 1:**
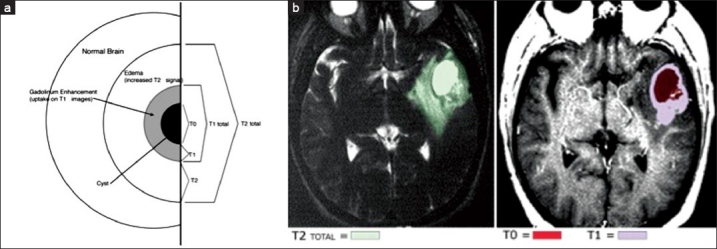
a) Schematic demonstrating the specific MR-imaging abnormalities analyzed in this study. b) Representative MR images with regions of imaging abnormality outlined (T0, T1 total and T2 total) demonstrate the method for quantitative tumor volume data collection

The definitions of terms used to identify specific imaging components, which apply to the remainder of the manuscript and the statistical analysis, are summarized in Table 2 and demonstrated in Figure 1a. Briefly, the volume of central necrosis represented as hypodensity on T1-weighted MR images is referred to as T0. The volume of tumor tissue that enhances with gadolinium on T1-weighted images is referred to as T1 (this does not include T0 if the patient has a central necrotic cyst). The volume of tissue that demonstrates increased T2-weighted signal intensity (excluding T0 and T1) is referred to as T2. When the total volume of gadolinium-enhancing tissue, including necrotic cysts, is analyzed, this volume is referred to as T1 total and is equivalent to T1+T0. The total volume of imaging abnormality encompassed by the region of increased T2 signal is referred to as T2total and is equivalent to T0+T1+T2. We also derived ratios for T2/T1 and T2/T1 total to determine whether the volume of edema alone (T2) relative to either the solid enhancing tumor component (T1) or the entire solid enhancing component, including the necrotic cyst (T1 total), predicted outcome.

**Table 2:** Variables used in statistical analysis

Clinical
Age
Sex
Preoperative Karnofsky performance score
Extent of surgical resection
Gross total resection
Sub-total resection
Biopsy
Adjuvant chemotherapy
Primary tumor location
Lobar
Deep
Mixed
Tumor Burden
T0- Necrotic cyst volume
T1- Gadolinium-enhancing volume excluding T0
T1 total- Gadolinium-enhancing volume including T0
T2- Edema/increased T2 signal volume excluding T1and T0
Midline shift
Invasiveness
T2 and T2 total volumes
Ratio T2/T1; T2/T1 total
Involvement of contralateral corpus callosum
Edema only
T1 gadolinium enhancement
Basal ganglia involvement
Edema only
T1 gadolinium enhancement
Brainstem involvement
Edema or T1 gadolinium enhancement
Subependymal contrast enhancement

### Quantitative imaging data acquisition

Preoperative T1-weighted gadolinium-enhanced and T2-weighted MR images were digitized as previously described.[10] Briefly, volumes for each tumor component (gadolinium enhancement, increased T2 signal or necrotic cyst) were calculated using National Institutes of Health NIH Image software(SCION Corporation). Volumes were calculated by summing the individual volumes of imaging abnormality represented by each slice. Slice volumes were calculated by multiplying slice areas by slice thickness. Area was calculated by tracing the outline of the imaging abnormality on each slice [Figure 1b]. The mean value from two independent observers’ calculations of T1 gadolinium enhancement and increased T2 signal was used for the analysis. Measurements of T1 volume were within 10% of midpoint values in 79% of cases, while measurements of T2 volume were within 10% of midpoint values in 70% of cases. The percent difference between the two observers’ T1 and T2 measurements, respectively, was 7.6% (±7.3) and 6.0% (±4.7). Maximum midline shift and central necrotic cyst volumes were measured by one observer and recorded. Because of its potential impact on MR image enhancement and increased T2 signal volumes, steroid use prior to imaging was recorded. Data were available for 67 of the 70 patients, of which 45 did not receive steroids and only 13 received steroids for greater than 48 hours prior to their imaging study.

### Qualitative imaging data

Qualitative features of preoperative imaging studies reflective of invasive tumor growth patterns were documented and recorded, including the presence of either T1 gadolinium enhancement or increased T2 signal involving the basal ganglia, corpus callosum (unilateral and contralateral) or brainstem; and the presence or absence of subependymal contrast enhancement. Subependymal contrast enhancement (SCE) was defined as the presence of any linear contiguous or noncontiguous gadolinium enhancement lining the ependymal surface. The side of the lesion and its location relative to deep versus lobar structures was recorded. Lesion location was recorded as primary involvement of deep or lobar structures or mixed involvement of both deep and lobar structures. Tumors were classified as primarily lobar (*n*= 58), deep (*n*= 6) or mixed deep and lobar (*n*= 8) based on the location of T1 gadolinium enhancement on preoperative MR images. Deep structures were considered to be the basal ganglia, thalamus, corpus callosum, septum, hypothalamus and brainstem. Brain regions outside these deep structures constituted a lobar location. A tumor was classified as deep if there was exclusive involvement of only deep structures. Tumors that appeared to originate outside deep structures were classified as primarily lobar, while those whose origin could not be identified as cortical or deep based on its anatomic location were defined as “mixed” in their location. Thus, designation as primarily lobar did not preclude secondary involvement of deep structures.

### Analysis of extent of resection

The extent of resection (EOR) was based on routine postoperative (within 48 hours) CT scans performed with and without contrast enhancement. The presence of nodular contrast enhancement on these postoperative CT scans defined the extent of resection as sub-total. Only cases with no evidence of nodular residual enhancement at the resection site (*n*= 32) were classified as gross total resections (GTRs). Seven patients underwent biopsy alone (BX), while 31 had a sub-total resection (STR).

### Statistical analysis

The association of tumor burden and invasiveness with patient survival was analyzed using a Cox multivariate backward stepwise model with significance level set at *P*= .05.[7] In addition to standard clinical variables (sex, age, Karnofsky performance score [KPS] at diagnosis), treatment-related factors that might have influenced overall survival, such as therapies after initial resection and radiation, were also included. Because of the sample size, not all the descriptive MR imaging variables were included in the statistical models. Those imaging features felt to identify an invasive phenotype most robustly (i.e., gadolinium enhancement involving the basal ganglia or crossing the corpus callosum and signal abnormalities in the brainstem) were included. It was assumed that T1 gadolinium enhancement or increased T2 signal that crossed to the contralateral corpus callosum represented the most extreme stage of tumor invasion in this location. Only 9 patients had either T1 gadolinium enhancement (*n*= 3) or T2 increased signal (*n*= 6) involvement of the brainstem, and they were grouped together to indicate brainstem involvement. The core variables included in the final statistical analysis are listed in Table 2. The relationship between individual imaging volume variables was analyzed with Pearson correlation analysis.

The multivariate statistical analyses were performed for the entire group of 70 patients, a subgroup of 52 patients in whom a gross total resection was deemed potentially anatomically feasible (GTR-eligible) and the subgroup of 32 patients who underwent actual GTR. The GTR-eligible subgroup was included to correct for bias introduced in the analysis of postoperative tumor burden and EOR by the differing tumor growth patterns in the STR and GTR groups. Thus, 11 of the 31 STR patients were excluded from the GTR-eligible subgroup because of the presence of gadolinium-enhancing tumor involving the contralateral corpus callosum, basal ganglia or other deep structures or brainstem. Since the extent of resection was based on the presence or absence of residual contrast-enhancing tissue, patients with increased T2 signal alone in the brainstem were not excluded (4 in GTR group and 3 in STR group). None of the 32 GTR patients exhibited any of the criteria used to exclude the 11 patients in the STR group. Differences in tumor volumes and ratios between patients with STR and GTR in the GTR-eligible subgroup were further analyzed using analysis of Variance(ANOVA). The actual GTR patients were studied separately to provide the most homogenous subgroup in which to analyze the impact of tumor volumes on outcome.

## RESULTS

### Quantitative analysis of tumor burden and midline shift

**Figure 2:**
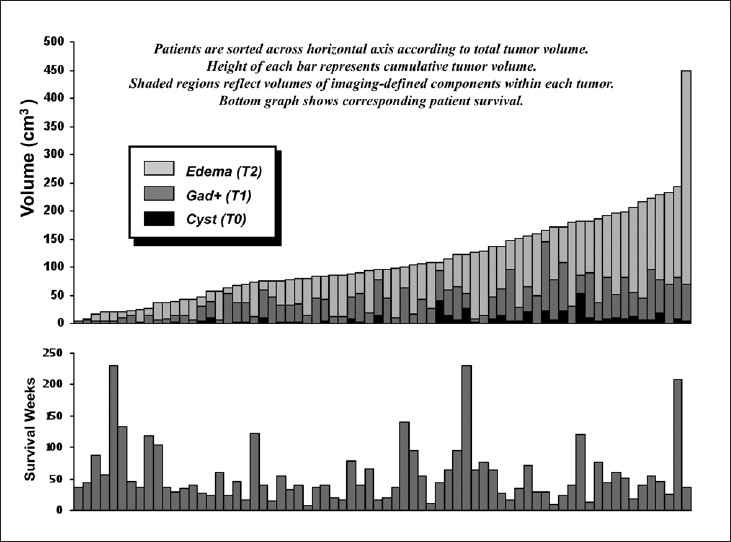
The histogram in the top panel demonstrates the variable contributions of central hypodensity (T0), gadolinium enhancement (T1) and increased T2 signal (T2) to overall tumor volumes. The corresponding overall survival of each patient is plotted in the histogram of the bottom panel. Note that no apparent relationship exists between tumor volume and overall survival as confirmed in Cox models (Table 4)

Volumetric analyses of imaging features demonstrated an extremely wide range of values [Table 1, Figure 2]. For the entire group, necrotic cysts were present in 39 patients with volumes ranging from 1.2 to 53.1 cm^3^. The volume of T1 gadolinium-enhancing tissue alone (T1) varied from 2.7 to 145 cm^3^ with a mean value of 42.6 cm^3^, while volumes of increased T2 signal alone (T2) ranged from 4.9 to 448.6 cm^3^ with a mean of 111.6 cm^3^. Midline shift, ranging from 0.2 to 1.6 cm, was found in 45 patients. To determin whether the volume parameters and ratios showed some inter-depdendence, we analyzed their correlations. Most measured volumes and ratios were significantly related (*P*< .05) with some exceptions [Table 3].

**Table 3:** Correlations among volume and ratio variables

	T1	T2	T1 total	T2 total	T2/T1	T2/T1 total
T0						
Pearson Correlation	0.391	0.087	0.635	0.335	– 0.231	– 0.323
Sig. (2-tailed)	0.001	0.475	0.000	0.005	0.054	0.006
T1						
Pearson correlation		0.305	0.959	0.645	– 0.484	– 0.506
Sig. (2-tailed)		0.010	0.000	0.000	0.000	0.000
T2						
Pearson correlation			0.283	0.916	0.343	0.306
Sig. (2-tailed)			0.018	0.000	0.004	0.010
T1 total						
Pearson correlation				0.644	– 0.477	– 0.524
Sig. (2-tailed)				0.000	0.000	0.00
T2 total						
Pearson correlation					0.074	0.025
Sig. (2-tailed)					0.543	0.838
T2/T1						
Pearson correlation						0.989
Sig. (2-tailed)						0.000

### Quantitative and qualitative analysis of invasiveness

**Figure 3:**
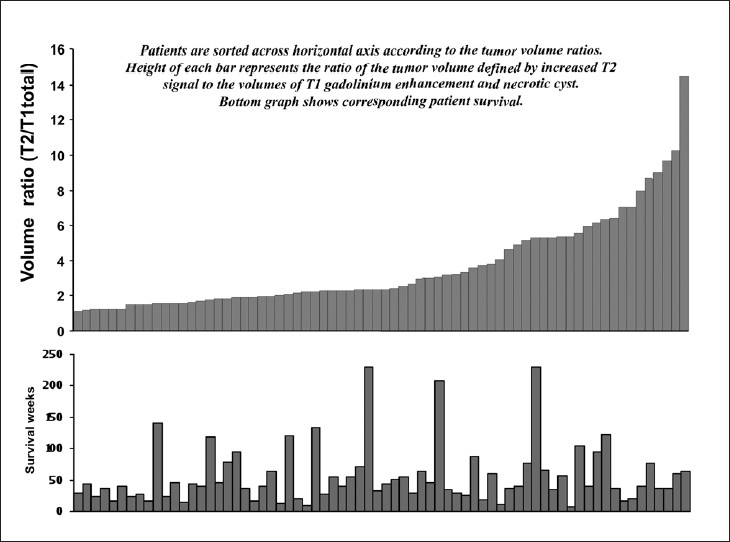
Histograms demonstrating the relationship between the ratio of T2 volume to T1 total volume (top panel) and survival (bottom panel). This ratio is presumed to allow comparisons between tumors of their propensity for infiltrative growth by normalizing T2 volumes to T1 total. The apparent lack of correlation between this ratio and survival was confirmed in Cox models (Table 4)

The region of increased T2 signal or edema (T2) on MRI contains ITCs[23,24] and provides a measure of ITC burden. The ratio of the volume of increased T2 signal or edema (T2) to the volume of enhancing tumor (T1), or T2/T1, is a potential measure, then, of the propensity for ITC infiltration in an individual tumor. The range of T2 and T2 total values is described in the preceding paragraph. The mean ratios of T2 to T1 total or T1 were 2.82 (±2.77) and 2.61 (±2.61), with ranges of (0.16 to 16.01) and (0.14 to 13.47), respectively, indicating extreme variability in the propensity for production of edema relative to volumes of enhancing tumor and necrotic cyst volumes. The variability in these ratio measurements and the corresponding patient survival are graphically displayed in Figure 3.

Qualitative analysis of invasive features, including tumor extension into deep structures or distant growth along white matter tracts such as the corpus callosum, was most informative for the group of primarily lobar tumors since mixed and deep tumors already had tumor involvement of deep or bilateral structures. Of the 58 primarily lobar tumors, 6 patients had gadolinium enhancement extending into the basal ganglia (*n*= 2), crossing the corpus callosum (*n*= 3) or both (*n*= 1). Of the remaining 52 lobar patients, 23 had increased T2 signal extending into the basal ganglia. Of 22 patients with imaging abnormalities in the corpus callosum, 15 had increased T2 signal alone limited to the ipsilateral corpus callosum, 3 patients displayed ipsilateral gadolinium enhancement and 4 patients had increased T2 signal crossing to the contralateral corpus callosum. Of these 4 patients, 1 had ipsilateral gadolinium enhancement. In 4 of these 52 patients, increased T2 signal was noted in the brainstem. In all cases, increased T2 signal equaled or exceeded the regions of T1 gadolinium enhancement.

Aside from involvement of the basal ganglia and corpus callosum, imaging abnormalities of the brainstem and the ventricular surfaces were analyzed as additional indicators of tumor spread. Overall, brainstem involvement was noted in 9 patients, 3 with gadolinium enhancement and 6 with increased T2 signal alone. All patients with gadolinium enhancement of the brainstem had deep tumors. Subependymal contrast enhancement (SCE) was noted in 23 patients, including 16 of 58 lobar tumors (28%) and 7 of 12 deep or mixed tumors (58%). Of the 52 patients in the GTR-eligible subgroup, 11 had SCE; and of these, 5 were classified as having an actual GTR based on the absence of nodular or bulk enhancing tumor at the primary tumor nidus on postoperative enhanced CT scans. Overall, only 19 of the 58 primarily lobar tumors lacked any of these imaging abnormalities felt to reflect tumor invasiveness.

### Correlation of tumor burden and invasiveness with survival

In a multivariate analysis of the entire group of 70 patients, variables associated with prolonged survival were younger age, preoperative Karnofsky score ≥ 70, lack of contralateral gadolinium enhancement in the corpus callosum and GTR. In a similar analysis of the GTR-eligible subgroup, prolonged survival was associated with younger age, greater midline shift, smaller T1-only gadolinium tumor volume and GTR versus STR. For the 32 patients with GTR, only age correlated with outcome, but the presence of edema in the contralateral corpus callosum was nearly significant (*P*= .068). The multivariate analysis of these groups is summarized in Table 4. The effect of corpus callosum invasion and GTR on outcome is represented graphically in a Kaplan-Meier plot [Figure 4]. Thus, for measures of tumor burden, the volume of gadolinium-enhancing tumor alone, or T1, for the GTR-eligible subgroup demonstrated a significant association with patient survival. For tumor invasiveness, gadolinium enhancement of the contralateral corpus callosum was the one feature associated with significant reduction in survival. A principal component analysis designed to integrate multiple measures of invasiveness (T2 only, T2only/T1only, invasion of basal ganglia, crossing corpus callosum, and brainstem involvement) did not demonstrate significant correlations between invasiveness and patient outcome when incorporated into CMVA (data not shown).

**Figure 4a:**
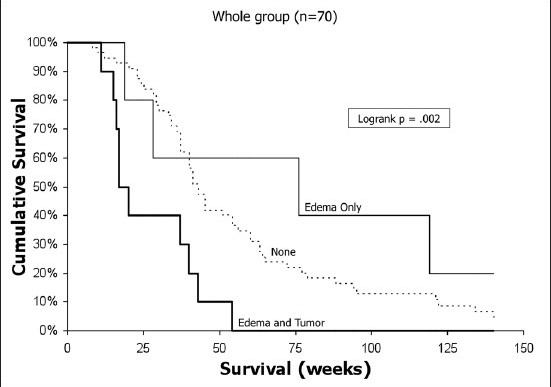
Kaplan-Meier survival curve demonstrates the impact of gadolinium enhancement crossing the corpus callosum in the total group of 70 patients. In the CMVA, this feature of tumor invasiveness predicted significantly shorter survival (*P*= .008; odds ratio, 3.14).

**Figure 4b:**
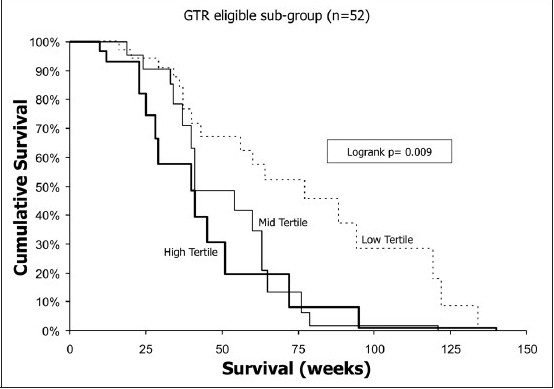
Kaplan-Meier survival curves demonstrating the impact of T1 volume (by tertile) on survival in the GTR-eligible subgroup

**Table 4:** Multivariate statistical analysis

Variable	Group/subgroup	Full (*n*=70)Odds		GTR-Eligible (*n*=52)Odds		GTR (*n*=32)Odds	
		Ratio	*P* value	Ratio	*P* value	Ratio	*P* value
Age (continuous)		1.64*	.000	1.63*	.000	1.76*	.001
Sex (M vs. F)			.403		.512		.944
KPS (<70 vs.≥70)		0.36	.007		.257		
EOR			.003				
	BX vs. GTR	4.01	.013				
	STR vs. GTR	3.12	.001	4.52	.000		
Other TX (#)			.533		.663		.858
Chemotherapy (Y/N )			.814		.532		.745
Primary location (Deep/mixed vs. Lobar)			.764		.942		.424
Midline shift (continuous)			.315	0.25	.015		.287
Basal ganglia			.748		.145		.507
	Edema only vs. None						
	Gad+ vs. None						
Contralateral corpus callosum			.015				
	Edema only vs. None	0.58	.303		.360		.068
	Gad+ vs. None	3.14	.008				
Subependymal C+ (Y/N)			.350		.264		.177
Brainstem involvement (Y/N)			.298				
T0 volume			.067		.890		.374
T1 volume			.753	1.02	.009		.723
T2 volume			.374		.773		.804
T2/T1			.531		.877		.886
T2/T1 total			.699		.862		.889
PCFA			.972		.580		.139

GTR: gross total resection; TX: treatment; T0: necrotic volume; STR: sub-total resection; Gad: gadolinium; T1: gadolinium-enhancing volume - T0; BX: biopsy C+: contrast enhancing; T2: increased T2 signal volume - T1 and T0; KPS: Karnofsky performance score; T1 total: T1 plus T0; *Odds ratio reported for a 10-year age differential; PCFA; principal component factor analysis

* Odds ratio reported for a 10-year age differential; PCFA; principal component factor analysis

Kaplan-Meier analysis of the effect of EOR on outcome showed nearly identical survival curves for STR and biopsy alone in the whole study group of 70 patients [Figure 5a] but significant differences between GTR and STR in both the whole group and the GTR-eligible subgroup [Figures 5a and 5b]. Thus, the GTR-eligible subgroup analysis corroborated the impact of GTR on survival seen in the total group. However, comparison of the tumor volume characteristics between the STR and GTR patients in the GTR-eligible subgroup revealed significant inter-group differences in tumor volumes for T2 (mean, 139.6 cm^3^ versus 91.05 cm^3^, respectively; *P*= .008), as well as T1 volumes in STR versus GTR patients (46.25 cm^3^ versus 33.56 cm^3^, respectively; *P*= .047) [Table 5]. Given that the T1 volume had an impact on patient survival in multivariate analysis for this subgroup, these differences in T1 volume between the actual GTR and STR patients may have contributed to the apparent differences in outcome related to EOR. The surprising finding of prolonged survival with *greater* midline shift in the GTR-eligible subgroup was further investigated by constructing Kaplan-Meier curves, which confirmed this relationship (data not shown).

**Figure 5a:**
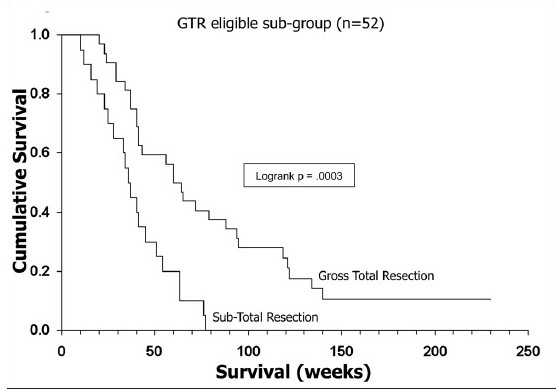
Kaplan-Meier analyses of the impact of EOR on survival for the entire study group (*n*= 70)

**Figure 5b:**
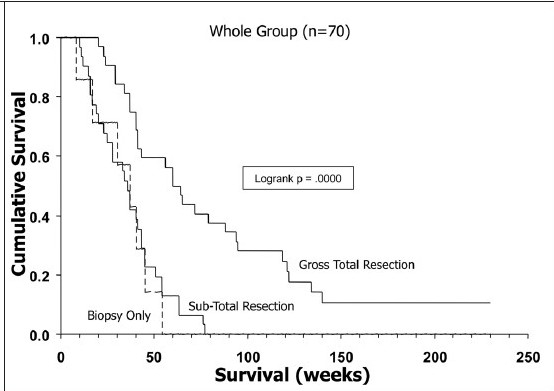
Kaplan-Meier analyses of the impact of EOR on survival for the GTR-eligible subgroup. Note that for the whole group, there is no difference in survival between the biopsy-alone and STR groups, but significant increase in survival for GTR patients in both groups

**Table 5:** Comparison of tumor volumes and ratios between STR and GTR groups

GTR-eligible subgroup					
Region	EOR	*n*	Mean (cm^3^)	Deviation (cm^3^)	ANOVA *P* value
T0	STR	20	5.45	5.94	
	GTR	32	5.91	11.16	0.868
T1	STR	20	40.79	22.19	
	GTR	32	27.65	22.95	0.047
T2	STR	20	93.3	45.63	
	GTR	32	57.5	45.11	0.008
T2/T1	STR	20	3.35	2.58	
	GTR	32	3.29	3.18	0.949

STR: sub-total resection; n: number; GTR: gross total resection

## DISCUSSION

This study tested the hypothesis that MR imaging features of untreated GBM patients that might reflect tumor burden and invasion would correlate with survival. This possibility is supported by the great variability in volume and distribution of gadolinium enhancement or increased T2 signal on MR images of GBM and the recognition that these features correspond histologically to solid tumor and isolated tumor cells (ITCs), respectively. Imaging phenotypes that correspond to outcome could improve patient stratification and provide a clinical discriminator to study GBM tumor biology. For instance, tumor samples from patients with distinct invasive imaging phenotypes could be used to analyze global gene expression profiles and elucidate the transcriptional basis for differential invasion. The feasibility of such a notion is evident in a report describing the identification of differentially expressed genes related to invasion in the T2 component of GBM compared with T1, such as insulin-like growth factor–binding protein 1 and matrix metalloprotease-2 expression.[9,18] In testing our hypothesis, we found that the invasive feature of gadolinium enhancement crossing the corpus callosum (for all 70 patients) and the tumor burden feature of greater T1 volume (for the GTR-eligible subgroup) were associated with shorter survival, while the lack of enhancing postoperative tumor burden predicted longer survival in both groups. However, no other measures of tumor burden or invasiveness, including invasive ITC burden (T2) or the ratio of this component to the solid gadolinium-enhancing tumor (T1) as an indicator of differential propensities for invasion, were associated with outcome. The interpretation and importance of these findings can best be understood by critical review of the assumptions about MR imaging and histologic correlations applied in this analysis.

For GBM, tumor burden should refer to the total number of tumor cells; but because of the diffusely invasive growth of GBM and the unknown limits for detection of ITCs by MR imaging, MR imaging–based estimates of GBM tumor burden must be restricted to determination of tumor “size” only. As noted in the introduction, MR imaging abnormalities in GBM correlate with distinct regional variations in cellular histologic composition. Thus, GBM patient biopsies from central hypodense cysts (T0), enhancing tumor (T1) and edema (T2) most consistently demonstrate necrosis, solid tumor with vascular proliferation and invasive isolated tumor cells, respectively.[11,23,24] These associations allow one to estimate histology-based tumor burden from MR images. Studies of patient biopsies cannot exhaustively sample all tissues with imaging abnormality, but correlation between near-terminal or terminal MR imaging and histopathology has confirmed that for untreated GBM, the region of T2 signal abnormality closely corresponds to the histologically identifiable extent of ITCs.[19,21,35]

An additional factor to consider is the extension of ITCs into radiographically normal brain. While ITCs clearly reside in brain that appears normal by imaging,[23,24,40] studies correlating postmortem histology and imaging indicate that the overall contribution of these cells to tumor burden is small.[19,21,35] Few attempts have been made to topographically map ITC densities, but those available suggest that total and proliferating tumor cell densities decrease as one travels away from the tumor epicenter,[8,35] with a more abrupt reduction in ITC density outside regions of increased T2 signal.[35] Thus, the present analysis fails to consider the population of ITCs residing in radiographically normal brain, but the existing data suggest that any error introduced by this limitation is likely to be small. The above studies support the concept of GBM as a gradient of tumor cells whose growth pattern is incompletely revealed by static MR images. As such, MR images of untreated GBM can estimate volumetric tumor burden of distinct histologic components but not tumor cell densities.

The significance of MR imaging features in GBM may extend to molecular programs with clinical relevance. Recent studies have identified GBM molecular sub-types with mesenchymal signatures that predict clinical behavior[5,38] which may in part be tied to enhanced invasiveness. Our lab has identified an important role for the pro-mesnenchymal gene, TWIST1, in GBM invasion through activation of properties similar to epithelial-mesenchymal transitions(EMT) associated with carcinoma invasion and metastasis[12,33]. Aghi *et al*. (2005) demonstrated an association in GBMs between increased T2/T1 ratios and epidermal growth factor overexpression EGFR overexpression,[1]. This molecular phenotype is associated with increased angiogenesis, edema and brain invasion.[29] all of which produce specific imaging changes. Of further interest, the link between EGFR mediated signaling and activation of TWIST1 in other cancers[31] supports the intriguing possibility that this pro-invasive signaling network may activate specific micro-environmental changes in invasive GBM that correlate with specific MR imaging features. While we did not find a survival change when stratifying patients according to T2/T1 ratios(or other indicators of invasiveness), the present study and that of Aghi *et al*.[1] suggest that imaging phenotypes should be studied alongside molecular phenotypes and that particular attention to imaging the ITC component may be useful in designing targeted therapy in the future.

A drawback of the present analysis is that static single images do not provide dynamic information on the rate of change for tumor burden and invasive features. It would be more informative to obtain serial MR images over time, but this is rarely clinically justifiable or practical in a patient with a new diagnosis of suspected GBM. For recurrent gliomas, one study of serial images demonstrated that solid tumor volume (i.e., T1) doubling times were more predictive of outcome than histologic tumor grade.[4] The impact of invasiveness on outcome for GBM patients has not been studied, but the direct *in vitro* correlation of glioma cell motility with invasiveness and tumor grade,[6,36] and individual differences in GBM tumor cell infiltration in brain slice culture studies[36] together suggest that the rate of glioma tumor cell invasion is a dynamic component of glioma biology that may contribute to patient outcome.

While our study did not allow dynamic analysis of invasive MR imaging features, we attempted to approximate potential differences in invasive propensity between tumors by analyzing the impact of the T2:T1 ratios on patient outcome. This analysis assumed that larger ratios of T2:T1 would indicate a greater propensity for ITC infiltration of normal brain when “normalized” to the T1 tumor volumes and thus provide a dynamic estimate of infiltrative behavior. However, this analysis alone or when combined with principal component analysis (PCFA) of other individual invasive related variables did not predict patient outcome. This negative result may reflect limitations in MR imaging to detect ITCs (see above), small differences in infiltration rates *in vivo*; or the possibility that while the infiltrative component of GBM is a major barrier to cure, it does not impact survival.

Despite these limitations, several important relationships of both tumor invasiveness and burden with survival were revealed. Of all the volume variables, only T1 volume in the GTR-eligible subgroup was associated with outcome. Only one other imaging variable, the presence of gadolinium-enhancing tumor crossing the corpus callosum, was associated with outcome in the total group of 70 patients. These patients were not included in the GTR-eligible subgroup analysis, where T1 volume reached significance. The GBM tumor growth patterns are characterized by a predilection for spread along white matter tracts,[15,30,39] and thus involvement of the corpus callosum is a potential robust indicator of invasive propensity. In fact, to our knowledge, in the only other study that specifically addresses corpus callosum involvement, the presence of any abnormal imaging (T1 or T2; ipsilateral or contralateral) defined two terminal nodes in patients less than 50 years old with KPS ≥70 with survival of 57 versus 105 weeks.[41] The selection of patients in the GTR-eligible subgroup eliminates the impact of gadolinium-enhancing tumor crossing the corpus callosum, and an effect of T1 volume becomes apparent. Most other studies do not report an impact of preoperative tumor volume or burden on outcome,[3,16,26,42,44] although there are exceptions.[13,20] In the GTR-only group, tumor volumes or invasiveness features did not influence outcome, possibly due to the small sample size or the power of age as a determinant of outcome in this group. Overall, the invasive feature of involvement of the contralateral corpus callosum, the brain’s largest white matter tract, predominated over tumor burden, suggesting that this feature of invasion provides a robust indicator of an invasive phenotype with prognostic implications.

Despite little impact of the various preoperative tumor volume measures on outcome, the absence of contrast enhancement was consistently associated with prolonged survival in both the total and GTR-eligible subgroups. This study does not conclusively establish the benefit of greater EOR, but it is of interest that the presence of any residual nodular contrast enhancement in the primary tumor bed conferred a distinct survival disadvantage, as was also pointed out mathematically by Woodward *et al*.[43] Of note, our data with median survival times (MSTs) of 61 versus 38 weeks for GTR versus STR/ BX, respectively, are strikingly similar to those of Albert *et al*.,[2] who employed the same EOR stratification (GTR= no residual contrast versus any volume for STR) and found MST >68 weeks versus 35 weeks for GTR versus STR/ BX, respectively.[2] T1 volumes were significantly larger for STR than GTR patients in the GTR-eligible subgroup [Table 5]; but in multivariate analysis, the contribution of GTR to improved outcome was statistically unrelated to preoperative T1 volumes. Thus, GTR may influence patient outcome through mechanisms related to the effects of residual tumor.

Analysis of the Kaplan-Meier survival curves suggests that the resection of all T1 material may have a biological impact aside from cytoreduction (actual or mathematical) that influences tumor growth. Survival curves for biopsy and STR patients were nearly identical, while significant increases were noted in survival for GTR patients. The solid or T1 portion of GBMs is a highly specialized microenvironment that conceivably drives tumor proliferation and spread, and which may function as such at low T1 tissue burdens present in some STR patients. For instance, extrapolationof data in the study by Nagashima *et al*.,[35] suggests that proliferative cell numbers are 13.7-fold and 28-fold higher in gadolinium-enhancing tissue than in regions of increased T2 signal or normal brain, respectively. In addition, in a study by Dalrymple,[8] the mean proliferative indices in contrast-enhancing tumor and peripheral hypodensity drop from 3.9% to 0.9%, respectively. We speculate that the T1 component of GBM provides a stimulus for tumor growth, possibly by supplying autocrine or paracrine local growth factors. Therefore, the benefits of complete removal of T1 (i.e., GTR) may be related to the growth lag required for less proliferative regions of residual edema to re-establish the T1 microenvironment and drive tumor growth at its previous rate. Taking proliferative potential into account, as well as other differences between T1 and T2 or imaging normal brain, such as tissue oxygenation, angiogenesis and cytokine production, the impact of complete resection of all enhancing tumor tissue may reflect the elimination of a distinct biological niche with enhanced proliferative potential that also facilitates tumor progression and provides a relatively resistant environment to therapy. The recognition in other cancers that tumor-stromal interactions are crucial to the malignant phenotype[34] may be relevant to considering the T1 microenvironment and its unique contributions to GBM biology which may impact survival as demonstrated here.

Additional findings of clinical interest include analysis of increased T2 signal, subependymal contrast enhancement (SCE) and midline shift. In the only other study that, to our knowledge, has analyzed the region of edema or T2 in GBM, Hammoud *et al*. found a quadratic relationship between increased T2 signal volume/gadolinium enhancement volume ratios and survival,[16] which was not evident in the present study. Subependymal enhancement was identified in 23 (33%) of our patients but was not found to have any prognostic significance. This is in agreement with one other study that specifically addressed the prognostic significance of SCE at initial diagnosis.[37] Our finding that greater midline shift was associated with better outcome in the GTR-eligible subgroup is contrary to the findings of previous studies evaluating this specific feature.[14] Its significance here is unclear; and although tempting to suggest that patients with greater shift had better resections, these two variables are independently significant in the multivariate analysis.

## CONCLUSIONS

In this study, we characterized the quantitative and qualitative variability of MR imaging features among GBMs at presentation. Our hypothesis that MR imaging features reflecting tumor invasiveness or burden would define clinically relevant phenotypes was supported by the findings that gadolinium enhancement crossing the corpus callosum (an invasive feature) predicted shorter survival in the unselected total patient group and that greater T1 tumor burden was associated with prolonged survival for the GTR-eligible subgroup of patients. In addition, the lack of detectable postoperative T1 tumor predicted longer survival in all groups, but biopsy and STR patients had similar median survival, suggesting a synergistic interaction between T1 and T2 tumor components that contributes to overall growth rates. The lack of association between other imaging features of invasiveness or ITC tumor burden and outcome may in part reflect the limitations in tumor cell detection by MR and the lack of dynamic information inherent in the evaluation of single–preoperative-imaging studies. Also, while the ITC component of GBMs theoretically limits their cure, the more robust impact of T1 solid tumor spread and volume on prognosis and the potential interaction between T1 and T2 tumor compartments in promoting growth, support the possibility that failure to control the T1 tumor component remains the predominant impediment to improved outcome for GBM. Future studies that can correlate imaging findings with particular molecular phenotypes will also be of use in planning targeted individualized therapy for GBM.
